# Comparative Feasibility Assessment of a Simplified Mossel EE–MacConkey Workflow for Detection of Enterobacteriaceae in Selected Non-Sterile Pharmaceutical Products

**DOI:** 10.1021/acsomega.6c02842

**Published:** 2026-10-13

**Authors:** Ana L. S. Bernardes, Luana A. F. Afiune, Osmar N. Silva, José Luís R. Martins, Antônio S. N. Aguiar, Lucimar P. Rosseto

**Affiliations:** † Biodiversity Research Laboratory, Evangelical University of Goiás, Anápolis 75083-515, Brazil; ‡ Laboratory of Pharmaceutical Sciences, Evangelical University of Goiás, Anápolis 75083-515, Brazil; § Laboratory of New Materials, Evangelical University of Goiás, Anápolis 75083-515, Brazil

## Abstract

Microbiological quality control of nonsterile pharmaceutical products requires reliable detection of Enterobacteriaceae while maintaining operational feasibility in routine laboratory settings. Harmonized pharmacopoeial methods involve sequential procedures, including pre-enrichment, selective enrichment, and differential isolation. These steps increase analytical time and resource requirements. In this study, a simplified culture-based workflow consisting of pre-enrichment in Tryptic Soy Broth, selective enrichment in Mossel Enterobacteria Enrichment broth, and isolation on MacConkey agar was comparatively evaluated against harmonized pharmacopoeial procedures. The assessment was performed using *Escherichia coli* (ATCC 8739) and *Salmonella typhimurium* (ATCC 14028) inoculated into finished pharmaceutical products (pravastatin sodium and erlotinib tablets) and a raw material (paracetamol). Qualitative recovery of target microorganisms was observed in the evaluated finished products under both workflows. Comparable qualitative recovery patterns were observed under the experimental conditions evaluated. The simplified approach reduced total analysis time from six to 4 days and decreased estimated culture media costs from USD 340.25 to USD 95.99 per analysis. No recovery of *E. coli* was observed in paracetamol samples under either workflow. Neutralization also failed to restore microbial growth, suggesting potential matrix-associated inhibitory effects. Within the defined experimental scope, the proposed approach proved practically applicable to the evaluated matrices. However, the study was limited to two finished pharmaceutical products and one pharmaceutical raw material. Therefore, broader applicability requires further evaluation across a wider range of pharmaceutical matrices.

## Graphical abstract

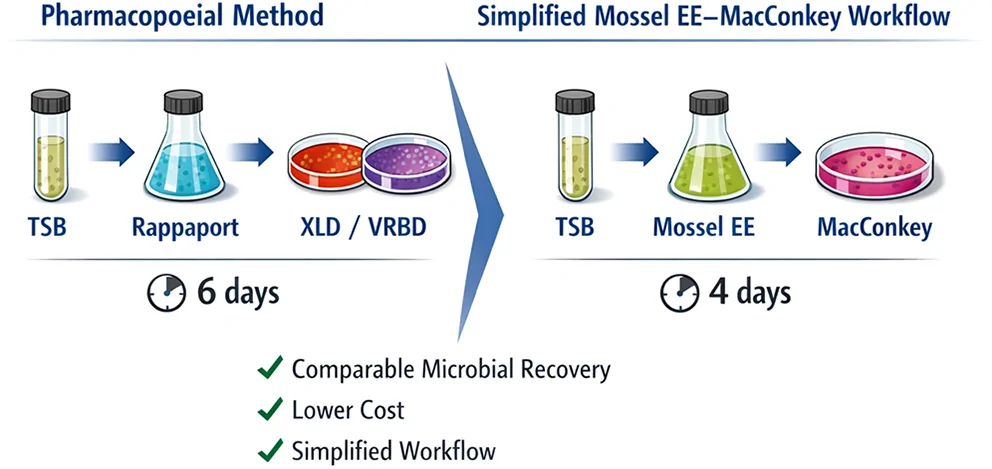

## 1 Introduction

Microbiological quality is essential to ensure therapeutic efficacy, patient safety, and product stability. Regulatory frameworks, including the United States Pharmacopeia (USP), the European Pharmacopoeia (EP), and the Brazilian Pharmacopoeia (FB), establish microbiological acceptance criteria for nonsterile pharmaceutical products, including limits for microbial contamination and the absence of specified microorganisms such as *Escherichia coli* and *Salmonella typhimurium*.^1–3^ In addition to defining microbiological acceptance criteria, these harmonized pharmacopoeias establish the essential microbiological requirements for examination of nonsterile pharmaceutical products, including sample preparation, nonselective pre-enrichment, selective enrichment, incubation conditions, microbiological suitability testing (Method Suitability Test), and interpretation of microbial growth. These harmonized procedures ensure that microbiological analyses are reproducible, comparable among laboratories, and capable of demonstrating that the pharmaceutical matrix does not interfere with microbial recovery.^1–3^

Despite implementation of Good Manufacturing Practices (GMP), microbiological contamination of active pharmaceutical ingredients (APIs), excipients, and finished pharmaceutical products continues to be reported.^4,5^ Such contamination may compromise product stability, therapeutic performance, and patient safety, particularly in susceptible populations.^6^ Culture-based microbiological methods remain the reference approach for microbiological quality control of nonsterile pharmaceutical products.^7–12^

Harmonized pharmacopoeial procedures involve sequential steps including nonselective pre-enrichment, selective enrichment, and isolation on selective or differential media. Although scientifically robust and internationally accepted, these procedures frequently require multiple culture media, parallel enrichment pathways, and extended incubation periods. Consequently, routine microbiological quality control may become operationally demanding, increasing analytical turnaround time, laboratory workload, and culture media consumption, particularly in laboratories operating under resource constraints.^10–12^

Recent advances in microbiological diagnostics, including chromogenic media, real-time polymerase chain reaction, and matrix-assisted laser desorption ionization-time-of-flight mass spectrometry (MALDI-TOF MS), have improved microbial detection and identification.^13–15^ However, these technologies require specialized instrumentation, trained personnel, and higher operational costs, limiting their routine implementation in many pharmaceutical quality control laboratories.^16–18^ Therefore, conventional culture-based methods remain the reference procedures for pharmacopoeial microbiological examination.

Selective enrichment using Mossel Enterobacteria Enrichment (EE) broth followed by isolation on MacConkey agar is widely employed for recovery and differentiation of Enterobacteriaceae in food, environmental, and clinical microbiology.^19–23^ However, its application as part of a simplified operational workflow for microbiological suitability testing of pharmaceutical products has received limited investigation.

Importantly, the present study does not modify any of the harmonized pharmacopoeial microbiological requirements. Rather, it evaluates whether the operational workflow can be simplified while preserving the essential microbiological principles established by the pharmacopoeias. Accordingly, the proposed workflow maintains the pharmacopoeial requirements for sample preparation, nonselective pre-enrichment, selective enrichment, incubation conditions, microbiological suitability testing (Method Suitability Test), and interpretation of microbial growth. The simplification investigated herein is restricted to the operational sequence of selective isolation and does not alter the microbiological acceptance criteria or the harmonized compendial requirements established by the USP, EP, and FB.^1–3^

Therefore, the objective of this study was to evaluate the practical feasibility of a simplified culture-based workflow consisting of pre-enrichment in Tryptic Soy Broth (TSB), selective enrichment in Mossel EE broth, and isolation on MacConkey agar for qualitative detection of Enterobacteriaceae in selected nonsterile pharmaceutical products inoculated with *E. coli* ATCC 8739 and *S. typhimurium* ATCC 14028. The study was designed to assess the operational applicability of this workflow under defined experimental conditions.

## 2 Experimental Section

### 2.1 Experimental Conditions

This study was conducted in collaboration with a private pharmaceutical company located in the Distrito Agro-industrial de Anápolis (DAIA), Goiás, Brazil. All experimental procedures were performed in restricted microbiological quality control laboratories equipped with qualified air-handling units (AHUs). The laboratories were classified as grade C, with grade D entry antechambers.

One pharmaceutical raw material and two synthetic oral pharmaceutical products in tablet form were analyzed. The finished products were provided by the manufacturers, whereas the raw material was obtained directly from the supplier. The evaluated samples included pravastatin sodium 40 mg tablets (Batch PLSC19023A; Aurobindo Pharma Limited, India; Dec 2019), erlotinib 150 mg tablets (Batch ER150002-B; Eugia Pharma Specialties Limited, India; Sep 2020), and paracetamol (Batch 1011726; Granule India Limited, Hyderabad, India; Jan 2022).

Thirty grams of each sample were tested in duplicate in accordance with the Brazilian Pharmacopoeia, sixth edition, Volume I, Section 5.5.3.1 (Microbiological Examination of Non-Sterile Products), which specifies duplicate plating for microbiological enumeration and testing procedures.^3^

The culture media used included TSB, Rappaport–Vassiliadis enrichment broth for *Salmonella*, Xylose Lysine Deoxycholate (XLD) agar, MacConkey agar, and Violet Red Bile Dextrose (VRBD) agar. All media were prepared in-house from dehydrated powder according to the manufacturer’s specifications. Mossel EE broth (Laborclin) was the only medium purchased ready-to-use.

All culture media underwent growth-promotion and sterility testing prior to use. Freeze-dried *E. coli* (ATCC 8739) and *S. typhimurium* (ATCC 14028) were used as test organisms. Soy lecithin (Anidrol) and polysorbate 80 (Dynamic) were used as neutralizing agents. All materials were sanitized, sterilized by autoclaving, or otherwise treated according to established laboratory procedures.

Microbial suspensions were standardized according to the Brazilian Pharmacopoeia,^3,6^ which recommends inocula not exceeding 100 colony-forming units (CFU) for microbiological method suitability testing. Lower inoculum concentrations (≤8 CFU/0.1 mL) were additionally evaluated to assess qualitative microbial recovery under low-inoculum conditions.

Bacterial cell counts for *E. coli* (ATCC 8739) and *S. typhimurium* (ATCC 14028) were determined by serial dilution using subcultures with fewer than five passages from the original ATCC stock cultures.^25–29^ For each microorganism, two plates were evaluated at each dilution level from 10^–6^ to 10^–10^. Following incubation at 32.5 ± 2.5 °C for 48 h, colonies were counted and the arithmetic mean was used to calculate CFU values. Lower inoculum concentrations (≤8 CFU/0.1 mL) were additionally evaluated to verify qualitative microbial recovery under low inoculum conditions.

### 2.2 General Microbiological Procedures

For qualitative evaluation, pre-enrichment was performed in nonselective TSB to facilitate recovery of stressed cells, followed by selective enrichment in appropriate broths and isolation on selective or differential media. Identification was performed using standard biochemical procedures. Sterile 90 × 15 mm Petri dishes were prepared under a biological safety cabinet by pouring 20 mL of molten agar, followed by incubation at 32.5 ± 2.5 °C for 72 h to confirm sterility. Microbiological testing was conducted according to pharmacopoeial protocols to assess method suitability: (a) Positive control: standard microbial suspension; (b) Product control: evaluation of intrinsic contamination; (c) Positive product control: evaluation of bacteriostatic activity; (d) Negative control: sterility verification of media and reagents; and (e) Positive media control: growth-promotion verification using standard microorganisms.

Samples were inoculated with microbial suspensions for positive controls. Negative and product controls were evaluated without inoculation. All procedures were conducted in compliance with Good Manufacturing Practices (GMP) requirements, ensuring data traceability and raw data integrity. The suitability of the microbiological analytical method was demonstrated through suitability testing, confirming compliance with acceptance criteria established by the Brazilian Pharmacopoeia and harmonized international guidelines.^3,6,29,30^ A comparative flowchart of the optimized method and the corresponding pharmacopoeial methods is provided in the Supporting Information (Supporting File S1).

For counting the total number of mesophilic microorganisms, 10 g of each sample were diluted (1:10) in 90 mL of TSB and incubated. Following incubation, 10 mL aliquots were transferred for further testing in accordance with the relevant pharmacopoeia monographs.^3,29,30^ Microbiological tests for the detection of bile-tolerant Gram-negative bacteria, *S. typhimurium*, and members of the Enterobacteriaceae family were conducted using harmonized pharmacopoeial procedures in parallel with a simplified workflow based on Mossel EE broth and MacConkey agar. The workflow included a pre-enrichment stage to facilitate the recovery of stressed microorganisms, followed by selective enrichment and differential isolation. For all approaches, samples were prepared by adding 10 g of product to 90 mL of TSB (1:10 dilution). For positive product controls, the standardized microbial suspension was added to the sample–broth mixture prior to incubation.

The pre-enrichment step consisted of incubation under conditions dependent on the target microorganism. Samples intended for bile-tolerant Gram-negative bacteria and for the simplified Mossel EE–MacConkey workflow were incubated at 22.5 ± 2.5 °C for 2–5 h. Samples intended for *S. typhimurium* detection were incubated at 32.5 ± 2.5 °C for 18–24 h, in accordance with pharmacopoeial recommendations. Following pre-enrichment, selective enrichment was performed using media appropriate for each analytical pathway. For the detection of bile-tolerant Gram-negative bacteria and for the simplified workflow, aliquots were transferred to Mossel EE broth (Step D) and incubated at 32.5 ± 2.5 °C for 24–48 h (Step E). This step allowed selective proliferation of Enterobacteriaceae. For *S. typhimurium*, enrichment was performed in Rappaport–Vassiliadis broth at the same temperature range for 18–24 h, promoting selective growth of the target pathogen. Subsequent isolation was performed on selective and differential agar media (Step F).

Violet Red Bile Dextrose (VRBD) agar was used for bile-tolerant Gram-negative bacteria, whereas Xylose Lysine Deoxycholate (XLD) agar was used for *S. typhimurium*. In the simplified workflow, MacConkey agar was used for isolation, enabling the simultaneous detection and presumptive differentiation of Enterobacteriaceae based on lactose fermentation patterns. After plating, all inoculated agar media were incubated at 32.5 ± 2.5 °C for 18–48 h (Step G), depending on the target microorganism and culture medium. Incubation conditions were selected to allow the development of characteristic colony morphology.

Results were interpreted based on colony morphology. On VRBD agar, red to violet colonies surrounded by bile precipitation halos were indicative of bile-tolerant Gram-negative bacteria. On XLD agar, red colonies with or without black centers were considered presumptive for *S. typhimurium*. On MacConkey agar, lactose-fermenting bacteria such as *E. coli* produced pink to violet colonies, whereas nonlactose fermenters, including *S. typhimurium*, produced colorless colonies.

The simplified workflow follows the same conceptual framework of pre-enrichment and selective enrichment but consolidates detection into a single isolation step using MacConkey agar. This approach reduces the number of culture media and incubation steps while maintaining the ability to differentiate major groups within the Enterobacteriaceae family. Sample preparation, incubation conditions, and interpretation criteria were consistent with those used in the optimized method described for Enterobacteriaceae detection. A schematic representation integrating the pharmacopoeial methods and the simplified Mossel EE–MacConkey workflow is presented in Figure 1.

**Figure 1:**
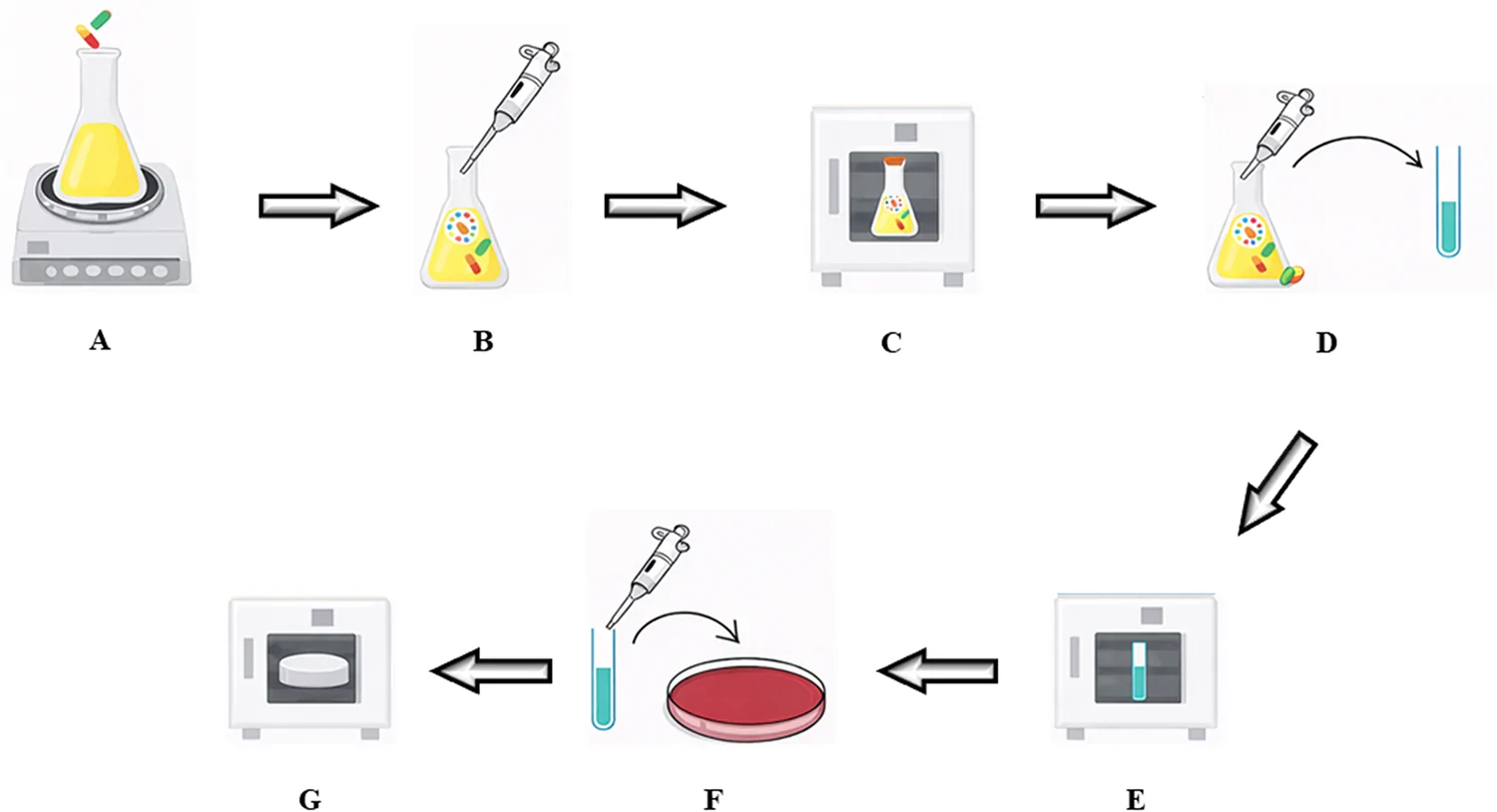
Integrated schematic representation of pharmacopoeial and simplified workflows for detection of Enterobacteriaceae and *Salmonella* species in nonsterile pharmaceutical products. The workflow is organized into sequential steps: (A) sample preparation by adding 10 g of product to 90 mL of TSB; (B) inoculation of the standardized working microorganism suspension for positive product controls; (C) pre-enrichment incubation under conditions dependent on the target microorganism; (D) transfer of aliquots to selective enrichment media; (E) incubation of selective enrichment cultures; (F) isolation on selective and differential agar media; (G) final incubation for colony development and interpretation.

### 2.3 Microbial Identification

Microbial identification was performed using the BBL Crystal Enteric system (BD), which incorporates chromogenic, fluorogenic, and conventional substrates. Colonies obtained from Tryptic Soy Agar (TSA) cultures were subjected to Gram staining, and indole and oxidase tests were performed. The resulting inoculum was loaded into the identification panels and incubated at 32.5 ± 2.5 °C for 18–24 h. Reaction profiles were subsequently analyzed using the BBL software for final identification.^7^

## 3 Results and Discussion

Microbial suspensions prepared by serial dilution yielded inoculum concentrations within the acceptance criteria established by harmonized pharmacopoeias for microbiological suitability testing (≤100 CFU/0.1 mL and ≤ 8 CFU/0.1 mL).^1–3^ The standardized inocula (Table 1) provided a consistent basis for qualitative comparison between the harmonized pharmacopoeial workflow and the simplified Mossel EE–MacConkey workflow under identical experimental conditions.

**Table 1:** Standardized Colony-Forming Unit (CFU) Counts of *E. coli* (ATCC 8739) and *S. typhimurium* (ATCC 14028) in Pharmaceutical Matrices at 10^–8^ and 10^–9^ Dilutions^a^

Microorganism	Dilution	Pravastatin Sodium (CFU/0.1 mL)	Erlotinib (CFU/0.1 mL)	Paracetamol (CFU/0.1 mL)
*E. coli*	10^–8^	79	56	60
(ATCC 8739)	10^–9^	5	5	8
*S. typhimurium*	10^–8^	70	23	68
(ATCC 14028)	10^–9^	6	4	5

a Values are expressed as CFU/0.1 mL.

Because the objective of this study was to evaluate the practical feasibility of a simplified microbiological workflow rather than to validate an alternative microbiological method, microbial recovery was assessed qualitatively following successful microbiological suitability testing and according to the acceptance criteria established by harmonized pharmacopoeias.^1–3,24,29,30^

Consequently, inferential statistical analyses and analytical validation parameters, including recovery efficiency, analytical sensitivity, robustness, reproducibility, and detection limits, were intentionally not evaluated.

Duplicate experiments were considered appropriate for this comparative feasibility study, which focused on qualitative microbial recovery under the same experimental conditions rather than on quantifying analytical variability or performing formal method validation. The graphical trends presented in Figure 2 are consistent with the colony-forming unit counts summarized in Table 1 and demonstrate the expected reduction in microbial counts across serial dilution levels. Together, these findings confirm adequate inoculum preparation and indicate that the experimental model was appropriate for comparative qualitative assessment of microbial recovery.

**Figure 2:**
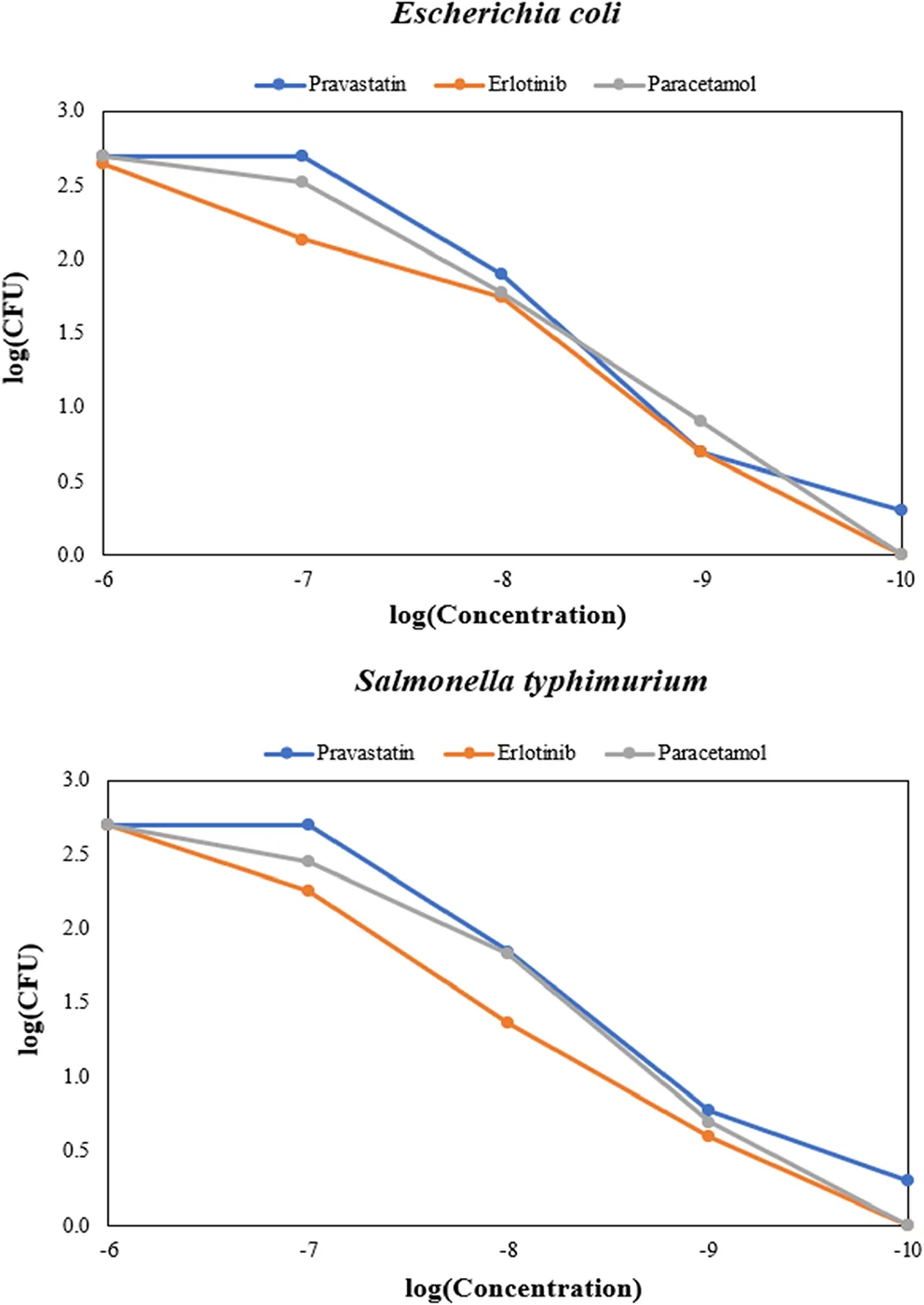
Descriptive analysis of microbial counts obtained from serial dilution assays for *E. coli* (ATCC 8739) and *S. typhimurium* (ATCC 14028) across different pharmaceutical matrices. Data are expressed as log (CFU) versus log­(concentration). Duplicate measurements showed similar trends across dilution levels, with counts falling within pharmacopoeial acceptance limits (≤100 CFU) at higher dilutions.

### 3.1 Pharmacopoeial Workflow

The harmonized pharmacopoeial workflow successfully recovered *E. coli* and *S. typhimurium* from the evaluated finished pharmaceutical products, demonstrating that the microbiological suitability requirements established before testing were achieved under the experimental conditions. Representative colony morphologies obtained on VRBD and XLD agar (Figures 3 and 4) were consistent with the expected phenotypic characteristics of Enterobacteriaceae and *Salmonella*, respectively.^1–3^

**Figure 3:**
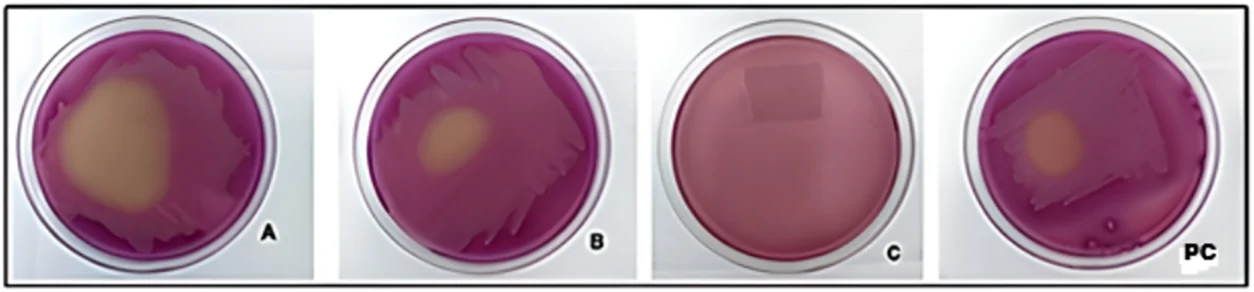
Qualitative recovery of bile-tolerant Gram-negative bacteria on Violet Red Bile Dextrose (VRBD) agar. (A) Pravastatin sodium 40 mg tablets; (B) erlotinib 150 mg tablets; (C) paracetamol raw material; (PC) positive control with *E. coli* (ATCC 8739).

**Figure 4:**
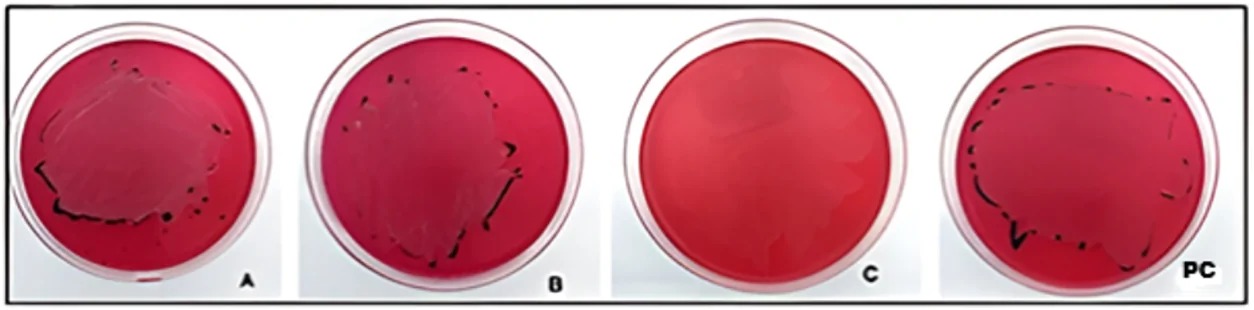
Qualitative recovery of *S. typhimurium* using the pharmacopoeial method on Xylose Lysine Deoxycholate (XLD) agar. (A) Pravastatin; (B) erlotinib; (C) paracetamol; (PC) positive control with *S. typhimurium* (ATCC 14028).

During selective enrichment, the observed color change in Mossel EE broth reflected microbial metabolic activity and selective proliferation of bile-tolerant Gram-negative bacteria. Subsequent isolation on VRBD agar yielded characteristic reddish-violet colonies surrounded by bile precipitation halos, while XLD agar produced red colonies with black centers typical of *S. typhimurium*. Identification using the BBL Crystal Enteric system confirmed the expected microorganisms, supporting the reliability of the microbiological procedures performed.^3,7^

The pharmacopoeial workflow served as the reference procedure against which the simplified workflow was comparatively evaluated, providing a benchmark for assessing whether simplification of the selective isolation stage could preserve qualitative microbial recovery under controlled laboratory conditions.

No recovery of either *E. coli* or *S. typhimurium* was observed in paracetamol samples using the pharmacopoeial procedure. Because the absence of recovery occurred before implementation of the simplified workflow, these findings suggest that the limiting factor was associated with characteristics of the pharmaceutical matrix rather than with the selective isolation strategy itself. This observation became particularly relevant when the simplified workflow produced the same outcome, reinforcing the hypothesis of matrix-associated interference rather than reduced analytical performance.

### 3.2 Simplified Mossel EE–MacConkey Workflow

The simplified workflow, consisting of pre-enrichment in Tryptic Soy Broth, selective enrichment in Mossel EE broth, and differential isolation on MacConkey agar, produced qualitative recovery patterns comparable to those obtained with the harmonized pharmacopoeial workflow for the evaluated finished pharmaceutical products.

Under the experimental conditions investigated, both *E. coli* (Figure 5) and *S. typhimurium* (Figure 6) were successfully recovered from pravastatin sodium and erlotinib tablets, indicating that simplification of the selective isolation stage did not compromise qualitative detection of the target microorganisms.

**Figure 5:**
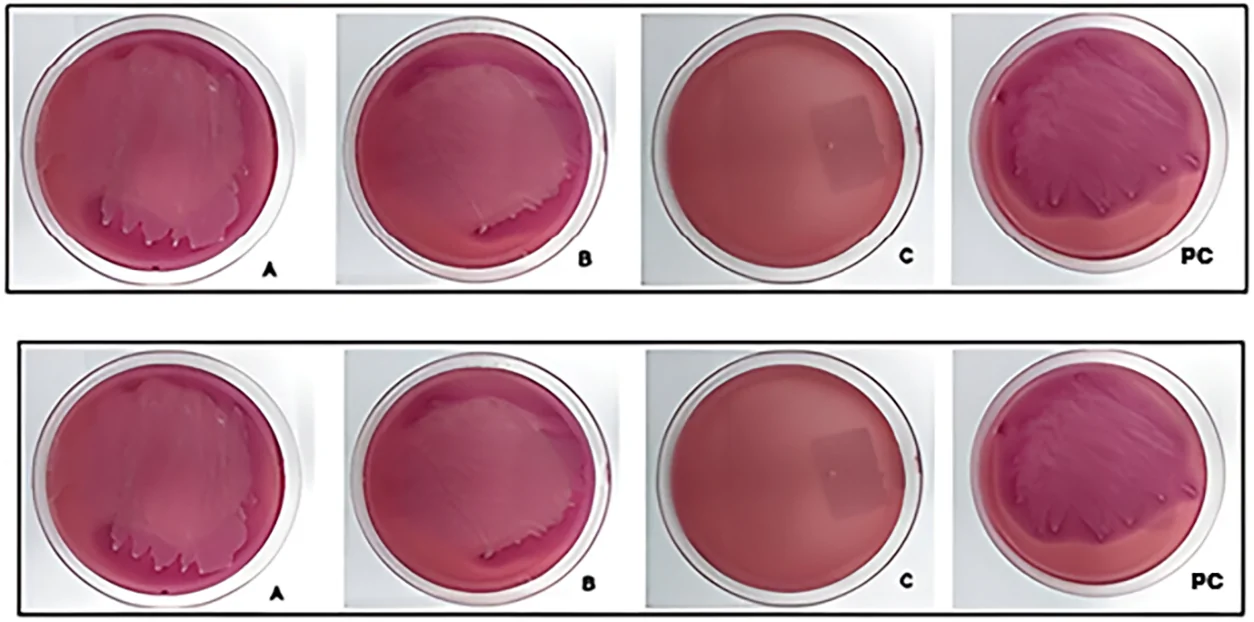
Qualitative recovery of *E. coli* (ATCC 8739) using the simplified Mossel EE–MacConkey agar workflow. (A) Pravastatin; (B) erlotinib; (C) paracetamol; (PC) positive control.

**Figure 6:**
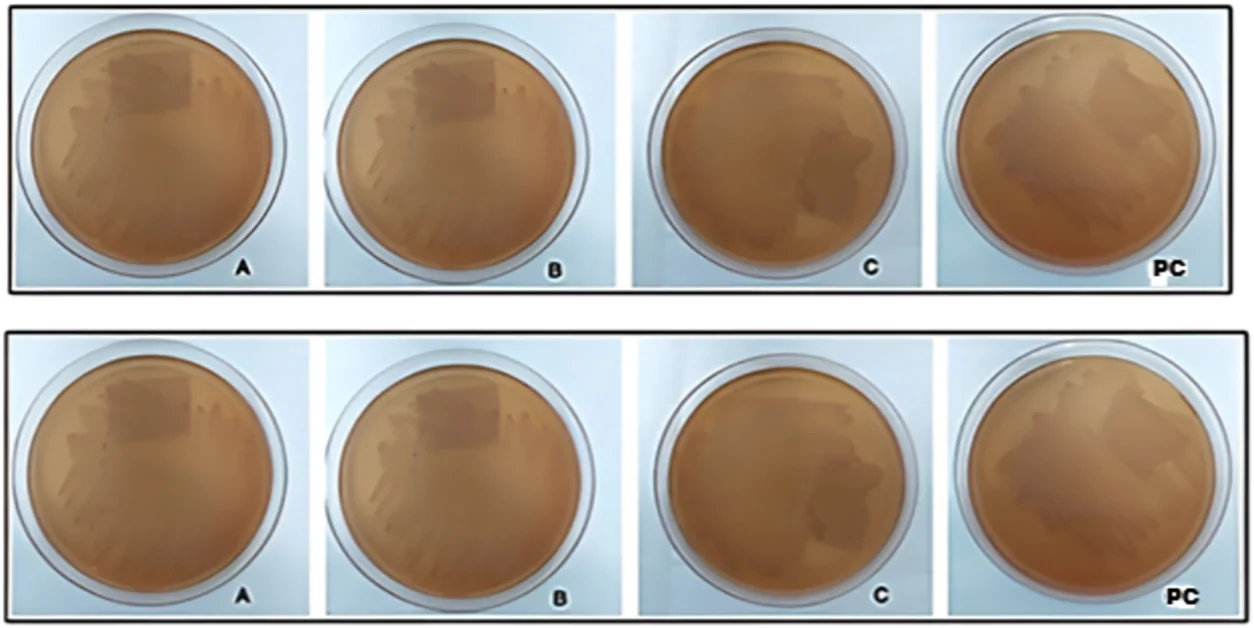
Qualitative recovery of *S. typhimurium* (ATCC 14028) using the simplified Mossel EE–MacConkey agar workflow. (A) Pravastatin; (B) erlotinib; (C) paracetamol; (PC) positive control.

The comparable performance observed between workflows can be explained by the fact that the proposed approach preserves the essential microbiological principles established by harmonized pharmacopoeias. Sample preparation, nonselective pre-enrichment, selective enrichment, incubation conditions, microbiological suitability testing, and interpretation of microbial growth remained unchanged throughout the study. The only modification investigated was the operational sequence of selective isolation, in which MacConkey agar was used as a single differential medium for qualitative detection of Enterobacteriaceae.^1–3,29,30^

MacConkey agar efficiently differentiated lactose-fermenting *E. coli*, which produced pink colonies, from nonlactose-fermenting *S. typhimurium*, which produced colorless colonies. These phenotypic characteristics are consistent with the well-established selective and differential properties of the medium and demonstrate that a single isolation medium was sufficient to discriminate the evaluated microorganisms under the defined experimental conditions.^19–23^

These findings should not be interpreted as validation or demonstration of equivalence to harmonized pharmacopoeial methods, which require comprehensive assessment of analytical performance characteristics before implementation of alternative procedures. Rather, the observed qualitative recovery provides evidence of the practical feasibility of the simplified workflow within the defined experimental scope.

### 3.3 Matrix Interference Assessment

The absence of *E. coli* recovery from paracetamol samples prompted additional investigation to determine whether the pharmaceutical matrix interfered with microbial recovery. Pre-enrichment was therefore repeated using Tryptic Soy Broth supplemented with 10% polysorbate 80 and 0.5% soy lecithin, neutralizing agents recommended by harmonized pharmacopoeias to minimize inhibitory effects during microbiological suitability testing.^1–3^ Neutralizer toxicity and growth promotion controls confirmed that the neutralizing system itself did not impair microbial recovery.

Despite these measures, *E. coli* was not recovered from the paracetamol matrix, whereas successful recovery was observed for the finished pharmaceutical products. Importantly, the same outcome was obtained using both the harmonized pharmacopoeial workflow and the simplified Mossel EE–MacConkey workflow, suggesting that the observed lack of recovery was associated with characteristics of the evaluated matrix rather than with the proposed simplification.

Previous studies indicate that paracetamol may interfere with bacterial physiology under specific experimental conditions. Seleem et al. demonstrated quorum sensing inhibitory activity, whereas Zimmermann and Curtis discussed evidence that nonantibiotic drugs, including paracetamol, may exhibit antimicrobial or bacteriostatic effects depending on the bacterial species, drug concentration, and experimental conditions.^11,12^ Nevertheless, these reports do not demonstrate that the absence of recovery observed in the present study resulted directly from the intrinsic antimicrobial activity of paracetamol.

According to harmonized pharmacopoeias, microbiological suitability testing is intended to demonstrate that the product matrix does not interfere with the detection of the specified microorganisms.^1–3^ Recovery may therefore be influenced by multiple factors associated with the pharmaceutical formulation, including the active pharmaceutical ingredient, excipients, physicochemical characteristics of the product, and the effectiveness of neutralization procedures.^1–3,10^ Consequently, the reduced recovery observed in this study should be interpreted as a matrix-dependent phenomenon rather than evidence of a specific inhibitory mechanism attributable exclusively to paracetamol.

These findings reinforce the importance of performing product-specific microbiological suitability testing before implementing routine microbiological analyses, particularly when evaluating alternative analytical workflows.^1–3^ Additional studies investigating different paracetamol concentrations, alternative neutralization systems, bacterial viability assays, and complementary microbiological approaches would be valuable to clarify the mechanisms responsible for the absence of *E. coli* and *S. typhimurium* recovery.

### 3.4 Operational Considerations

As summarized in Table 2, simplification of the selective isolation stage resulted in measurable reductions in both culture media consumption and overall analytical turnaround time. Under the experimental conditions of the present study, the estimated culture media cost decreased from USD 340.25 to USD 95.99 per analysis, while the total analytical time was reduced from six to 4 days.

**Table 2:** Comparative Culture Media Costs per Analysis for the Pharmacopoeial and Simplified Workflows (USD)^a^

Culture Medium	Pharmacopoeial workflow (USD)	Simplified Workflow (USD)
Tryptic Soy Broth (TSB)	34.26	34.26
Mossel EE Broth	6.53	6.53
Rappaport Broth	126.00	—
MacConkey Broth	83.75	—
MacConkey Agar	55.20	55.20
XLD Agar	83.40	—
VRBD Agar	55.80	—
**Total per Analysis**	**340.25**	**95.99**

a Costs were calculated based on laboratory-scale acquisition volumes and include culture media only.

The reported cost comparison was restricted to culture media. Labor, general laboratory consumables, equipment-related expenses, and other operational costs were not included in the monetary estimates.

These findings have practical relevance for pharmaceutical microbiology laboratories because culture media represent a recurring operational resource in routine microbiological quality control. Reducing the number of culture media required for analysis may decrease material consumption, simplify media preparation and handling, reduce storage requirements, and improve laboratory workflow efficiency, particularly in laboratories performing high numbers of microbiological analyses.

However, the magnitude of the observed cost and time reductions should be interpreted with caution. The estimates presented in this study were calculated using the purchasing costs, workflow organization, and operational conditions of the participating laboratory. Consequently, the absolute economic benefit and analytical turnaround time may vary considerably among laboratories depending on factors such as local procurement policies, media suppliers, analytical throughput, and institutional practices.

## 4 Conclusions

This study evaluated a simplified workflow based on Mossel EE broth followed by MacConkey agar plating for the qualitative detection of *E. coli* (ATCC 8739) and *S. typhimurium* (ATCC 14028), two representative species commonly used in pharmacopoeial microbiological testing. Under the evaluated experimental conditions, qualitative recovery of the target microorganisms was observed in pravastatin sodium and erlotinib tablet matrices. Compared with harmonized pharmacopoeial procedures, the simplified workflow required fewer culture media and reduced analytical time from six to 4 days, resulting in lower estimated culture media costs.

These operational advantages were achieved while maintaining qualitative recovery of the evaluated microorganisms in the tested finished products. Recovery of *E. coli* was not observed in paracetamol samples, even after incorporation of polysorbate 80 and soy lecithin as neutralizing agents. Because similar findings were obtained using the pharmacopoeial procedure, the absence of recovery appears to be more closely associated with matrix-related effects than with limitations of the simplified workflow. This finding highlights the importance of product-specific suitability assessment, particularly for pharmaceutical matrices with potential inhibitory characteristics.

Overall, the simplified Mossel EE–MacConkey workflow provides a practical option for microbiological suitability testing of the evaluated pharmaceutical matrices under the defined experimental conditions. Further studies involving a broader range of pharmaceutical matrices, including liquid and semisolid dosage forms, are warranted to establish their wider applicability.

## Supplementary Material



## Notes

Flowchart of the optimized method for the detection of Enterobacteriaceae and corresponding pharmacopoeial methods (bile-tolerant Gram-negative bacteria, *Salmonella* spp., and *E. coli*) (Figure S1) (PDF)
