# Supplementary material for: Comparative Feasibility Assessment of a Simplified Mossel EE–MacConkey Workflow for Detection of Enterobacteriaceae in Selected Non-Sterile Pharmaceutical Products
Source: ACS Omega. 2026 Oct 13;11(42):64465–72. doi: 10.1021/acsomega.6c02842 (PMC13717654; doi:10.1021/acsomega.6c02842)

Supporting Information

Comparative Feasibility Assessment of a Simplified Mossel EE–MacConkey Workflow for Detection of Enterobacteriaceae in Selected Non-Sterile Pharmaceutical Products

Ana L. S. Bernardes[1], Luana A. F. Afiune[1], Osmar N. Silva[2], José Luís R. Martins[2], Antônio S. N. Aguiar[3], and Lucimar P. Rosseto[1,2]*

[1] Biodiversity Research Laboratory, Evangelical University of Goiás (UniEVANGÉLICA), Anápolis, Goiás, Brazil

[2] Laboratory of Pharmaceutical Sciences, Evangelical University of Goiás (UniEVANGÉLICA), Anápolis, Goiás, Brazil

[3] Laboratory of New Materials, Evangelical University of Goiás (UniEVANGÉLICA), Anápolis, Goiás, Brazil

Figure S1. Flowchart of the optimized method for the detection of *Enterobacteriaceae* and corresponding pharmacopoeial methods (bile-tolerant Gram-negative bacteria, *Salmonella* spp. and *Escherichia coli*).

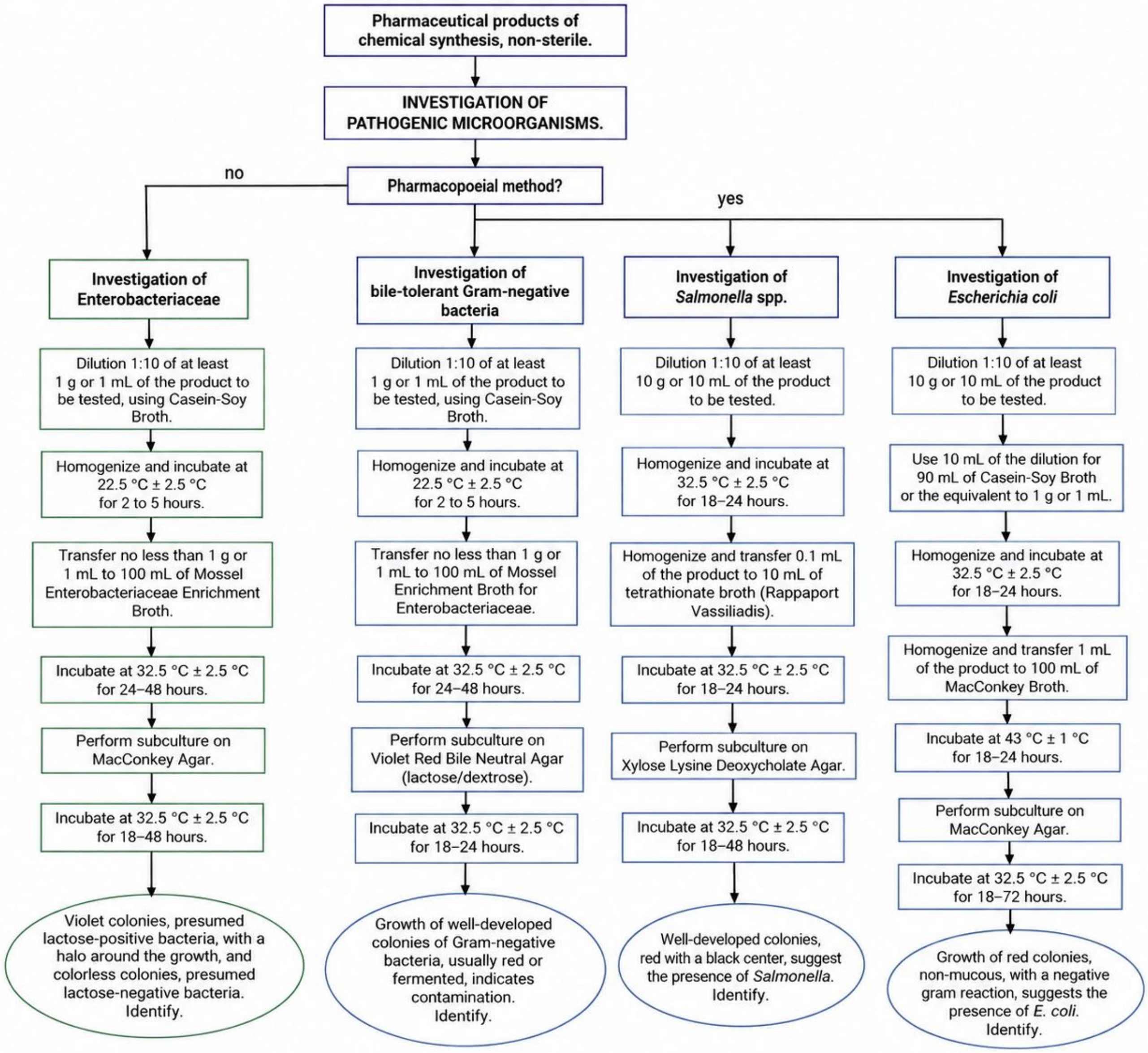

Supplement: Supplementary file 1 [file ao6c02842_si_001.pdf]
